# Supplementary material for: Potent Antiviral Activity against HSV-1 and SARS-CoV-2 by Antimicrobial Peptoids
Source: Pharmaceuticals (Basel). 2021 Mar 31;14(4):304. doi: 10.3390/ph14040304 (PMC8066833; doi:10.3390/ph14040304)

Supplemental information

Supplemental figure S1. LC/MS traces of peptoids

LC / MS trace of MXB1. t_R = 3.91 min. Gradient: 5–95% acetonitrile + 0.1% TFA over 6.80 min. MS (ESI$^+$, [M]$^+$, $C_{104}H_{139}N_{17}O_{12}$): 1820.70.

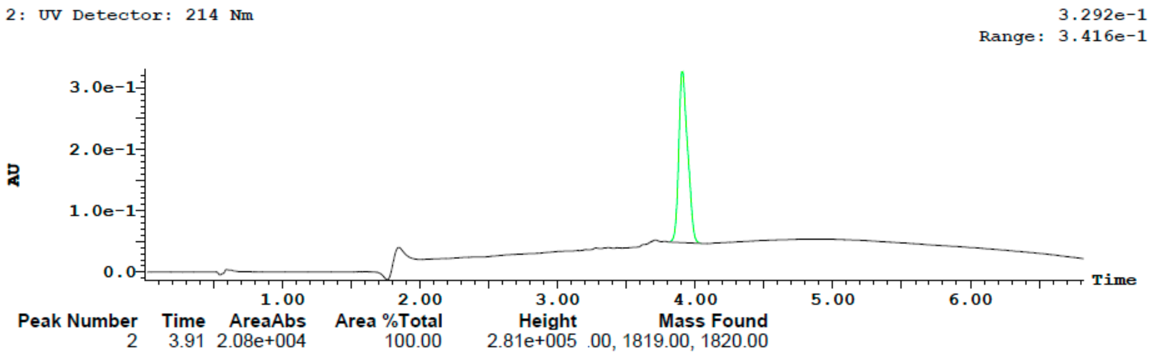

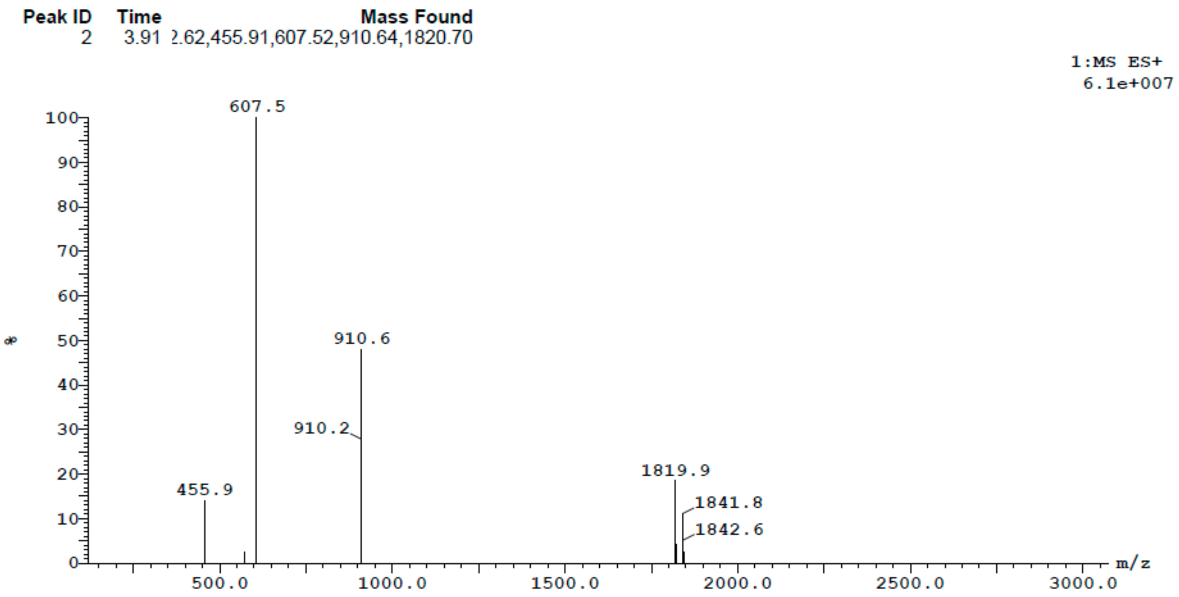

LC / MS trace of MXB2. t_R = 3.84 min. Gradient: 5–95% acetonitrile + 0.1% TFA over 6.80 min. MS (ESI^+, $[M]^+$, $C_{52}H_{69}Br_2N_9O_6$): 1074.41.

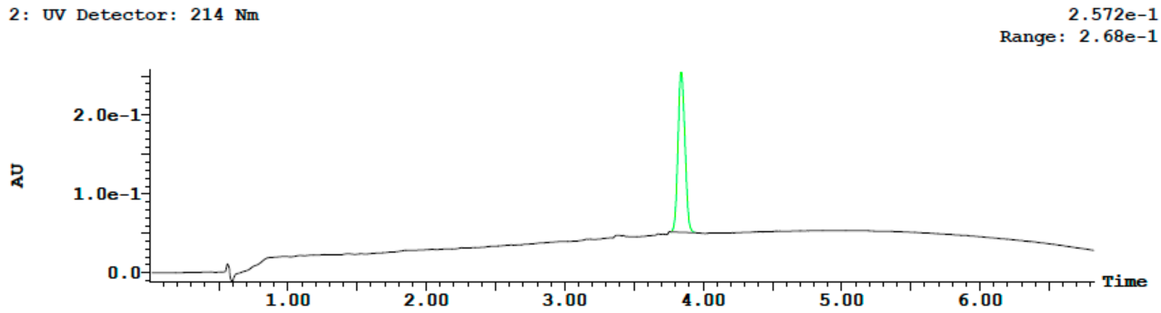

Peak Number	Time	AreaAbs	Area %Total	Height	Mass Found
2	3.84	1.19e+004	100.00	2.06e+005	.00, 1074.00, 1075.00

Peak ID	Time	Mass Found
3	3.21	0,1074.41,1098.24,538.80,1076.40

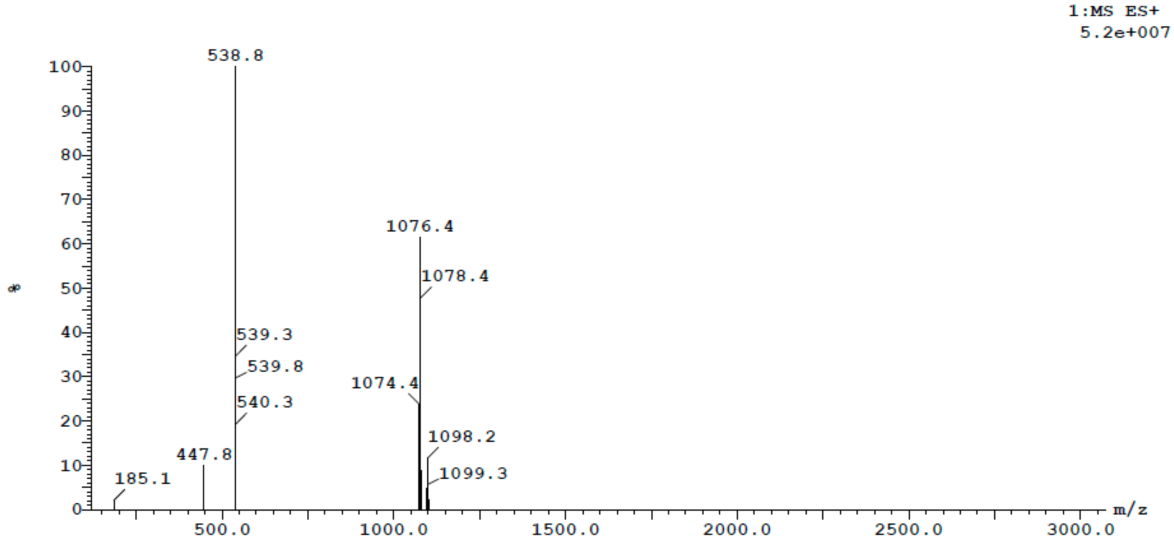

LC / MS trace of MXB3. t_R = 3.59 min. Gradient: 5–95% acetonitrile + 0.1% TFA over 6.80 min. MS (ESI$^+$, [M]$^+$, $C_{52}H_{70}BrN_9O_6$): 996.50.

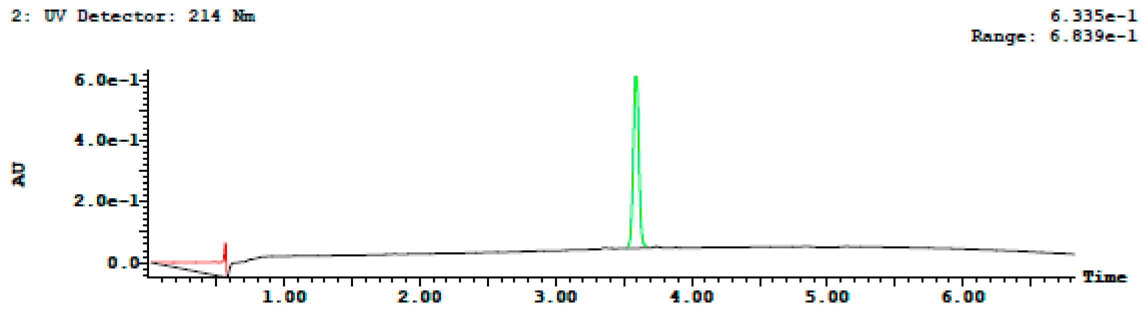

Peak Number	Time	AreaAbs	Area %Total	Height	Mass Found
1	0.57	1.48e+004	35.10	1.15e+005	Not Found
2	3.59	2.74e+004	64.90	5.86e+005	95.00, 996.00, 997.00

Peak ID	Time	Mass Found
2	3.60	9.50,498.95,1020.46,499.83,998.46

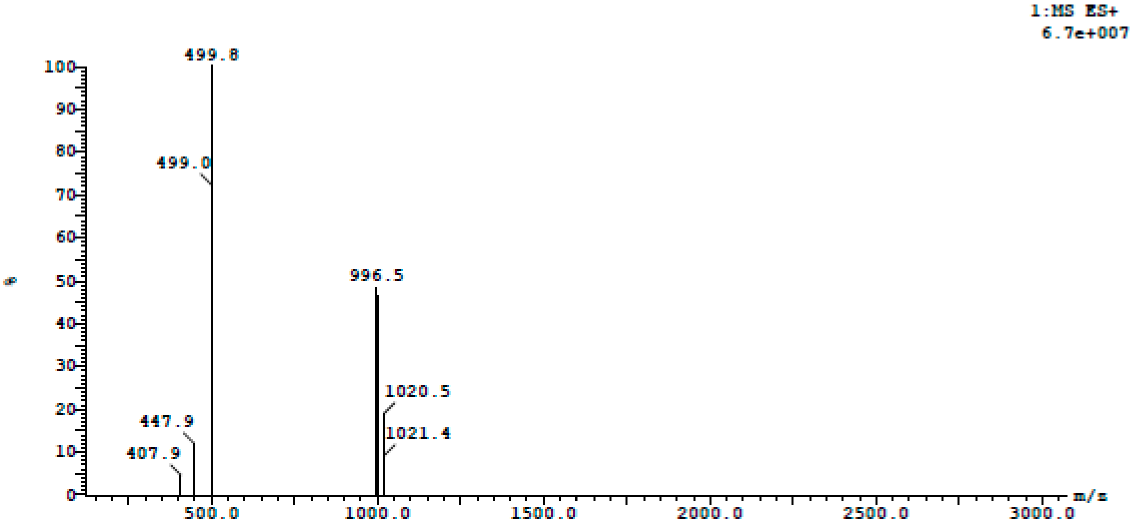

LC / MS trace of MXB4. t_R = 4.02 min. Gradient: 5–95% acetonitrile + 0.1% TFA over 6.80 min. MS (ESI$^+$, [M]$^+$, $C_{52}H_{67}Br_4N_9O_6$): 1233.09.

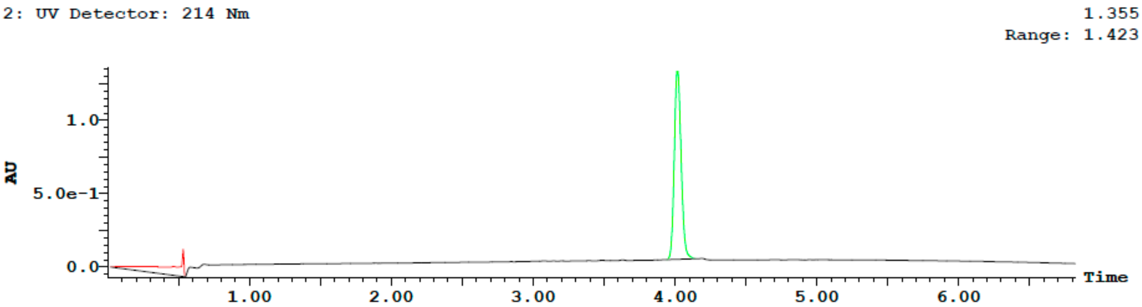

Peak Number	Time	AreaAbs	Area %Total	Height	Mass Found
1	0.53	1.88e+004	20.63	1.86e+005	Not Found
2	4.02	7.22e+004	79.37	1.30e+006	1231.00, 1233.00

Peak ID	Time	Mass Found
2	3.49	[illegible]2,1233.09,1256.11,617.70,1234.19

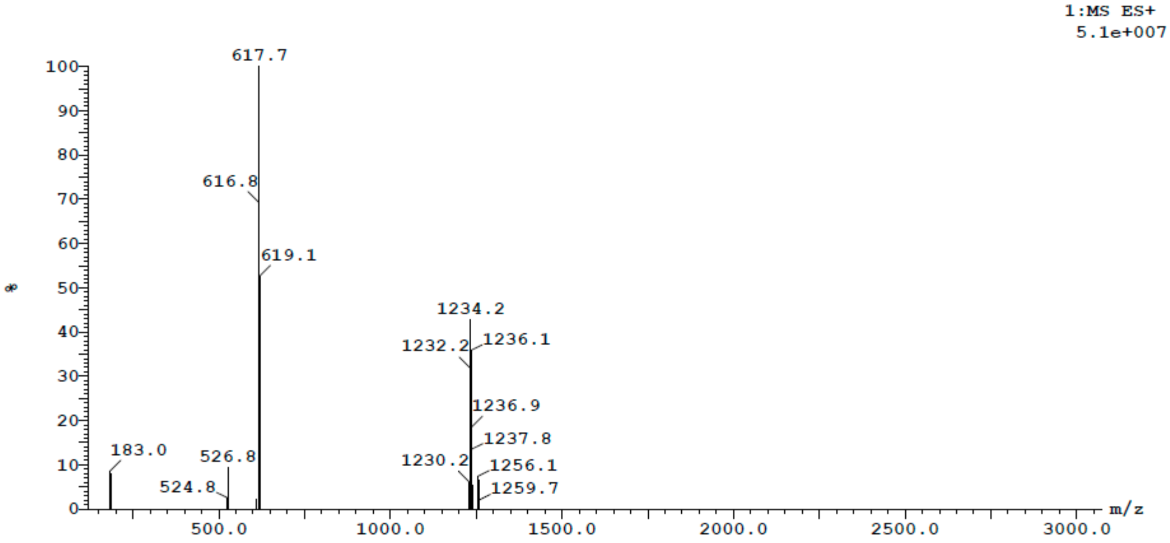

LC / MS trace of MXB5. t_R = 3.36 min. Gradient: 5–95% acetonitrile + 0.1% TFA over 6.80 min. MS (ESI$^+$, [M]$^+$, $C_{47}H_{78}N_8O_5$): 835.65.

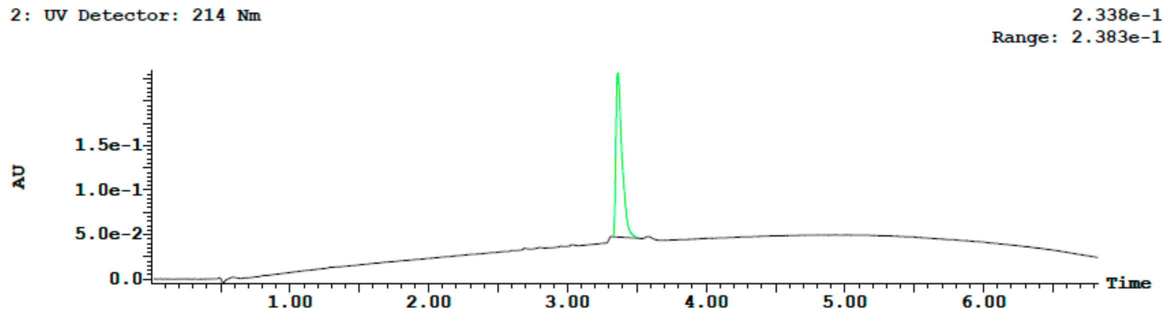

Peak Number	Time	AreaAbs	Area %Total	Height	Mass Found
1	3.36	9.50e+003	100.00	1.87e+005	834.00, 835.00

Peak ID	Time	Mass Found
7	3.39	35.65,858.53,279.35,418.86,836.68

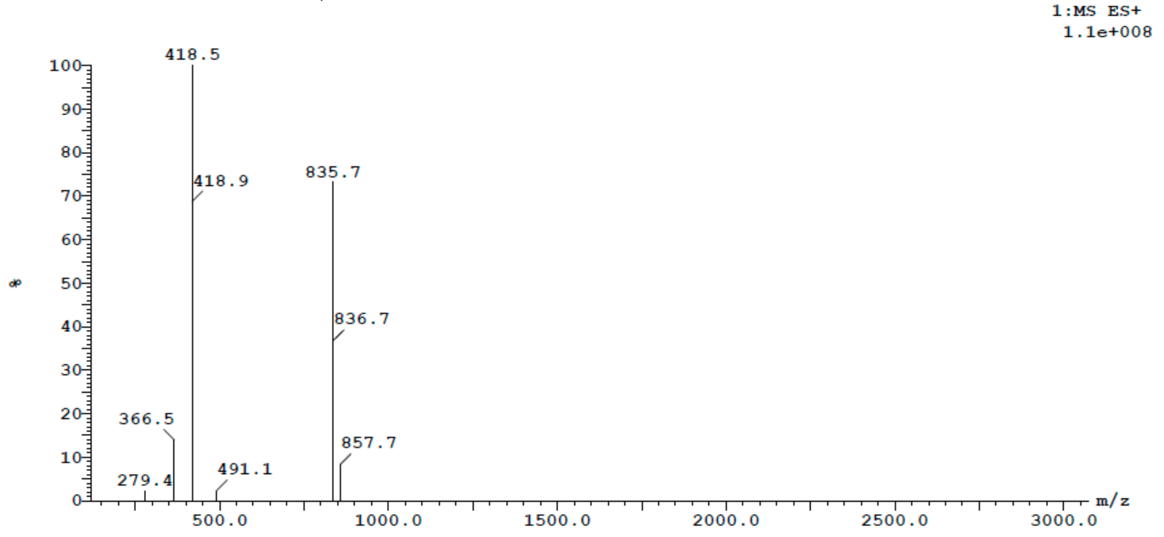

LC / MS trace of MXB6. t_R = 3.39 min. Gradient: 5–95% acetonitrile + 0.1% TFA over 6.80 min. MS (ESI$^+$, [M]$^+$, $C_{94}H_{128}N_{16}O_{11}$): 1658.67.

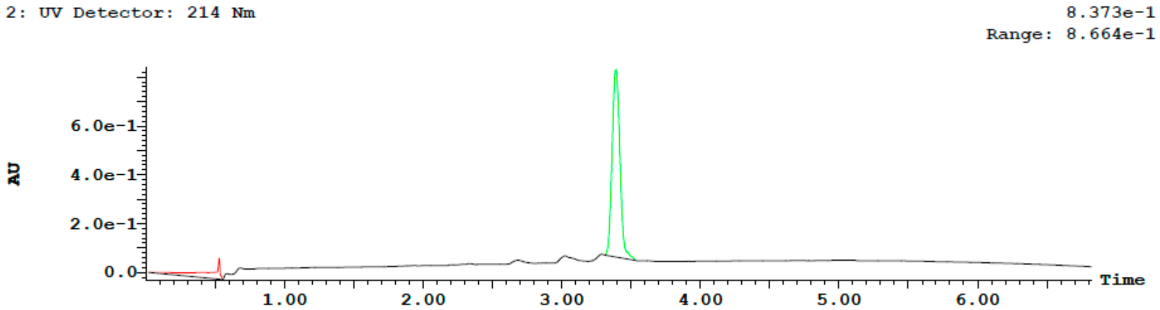

Peak Number	Time	AreaAbs	Area %Total	Height	Mass Found
1	0.53	7.94e+003	13.60	8.67e+004	Not Found
6	3.39	5.05e+004	86.40	7.74e+005	1657.00, 1658.00

Peak ID	Time	Mass Found
6	3.40	).74,415.46,553.64,829.60,1658.67

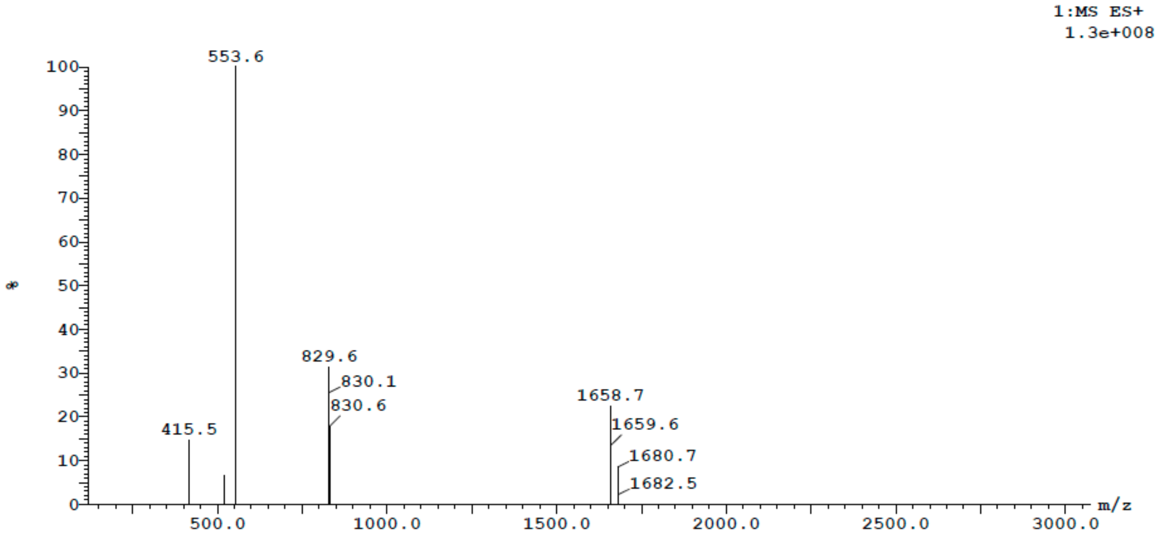

LC / MS trace of MXB7. t_R = 3.08 min. Gradient: 5–95% acetonitrile + 0.1% TFA over 6.80 min. MS (ESI^+, $[M]^+$, $C_{52}H_{71}N_9O_6$): 918.61.

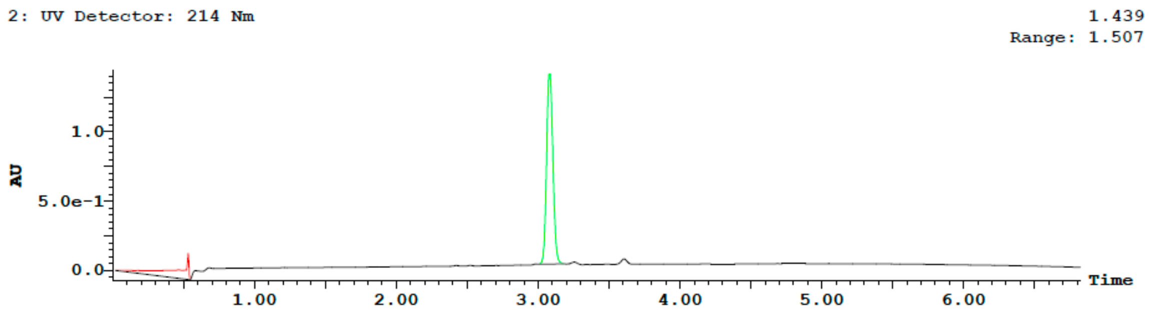

Peak Number	Time	AreaAbs	Area %Total	Height	Mass Found
1	0.53	1.88e+004	20.86	1.89e+005	Not Found
4	3.08	7.14e+004	79.14	1.39e+006	917.00, 918.00

Peak ID	Time	Mass Found
4	3.09	459.68,940.60,459.68,918.61

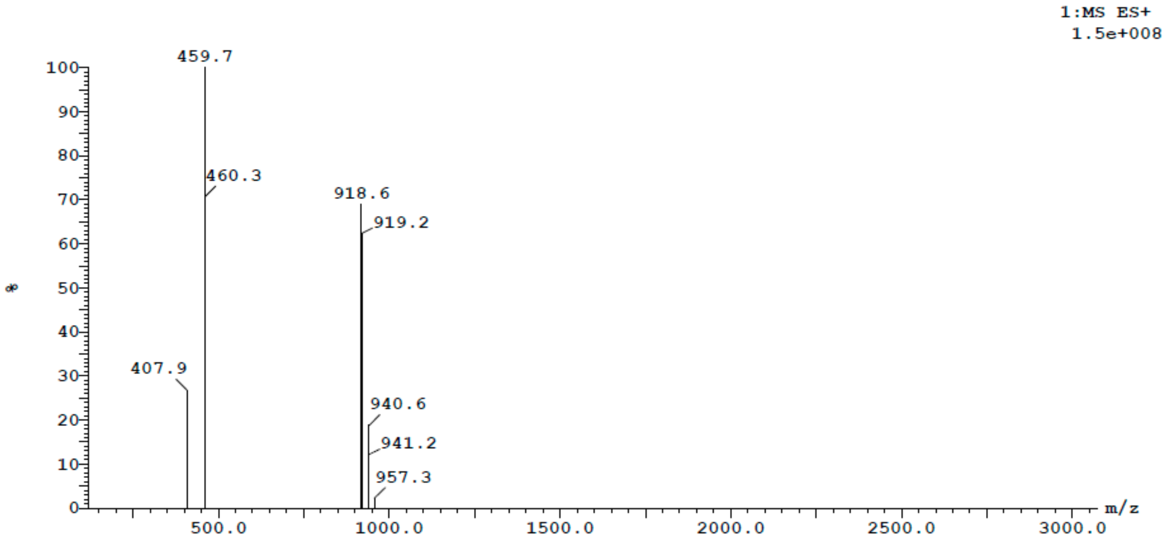

LC / MS trace of MXB8. t_R = 3.72 min. Gradient: 5–95% acetonitrile + 0.1% TFA over 6.80 min. MS (ESI$^+$, [M]$^+$, $C_{64}H_{94}N_{10}O_7$): 1115.74.

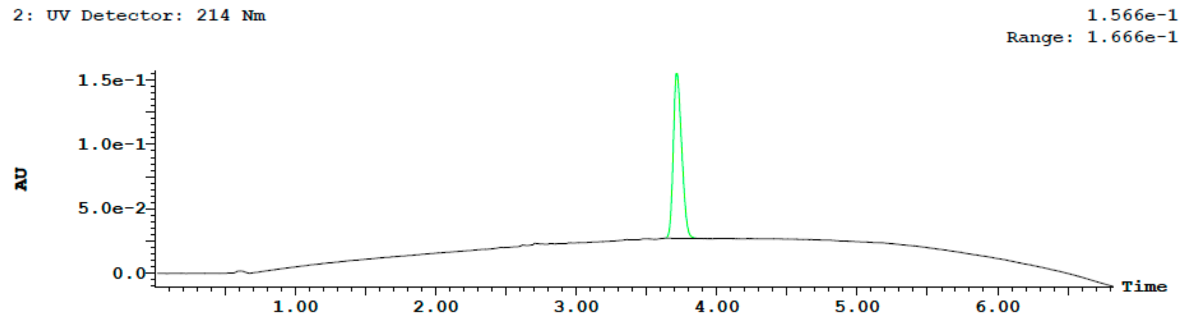

Peak Number	Time	AreaAbs	Area %Total	Height	Mass Found
2	3.72	8.90e+003	100.00	1.29e+005	[illegible].00, 1115.00, 1116.0[illegible]

Peak ID	Time	Mass Found
2	3.73	[illegible].51,1115.74,372.65,558.51,1116.7[illegible]

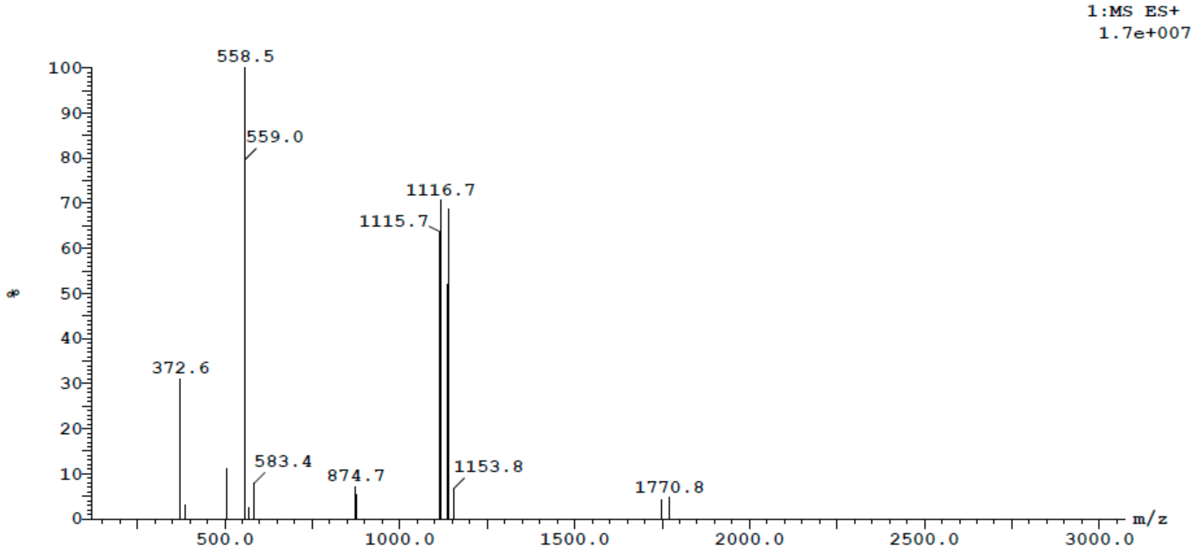

LC / MS trace of MXB9. t_R = 3.94 min. Gradient: 5–95% acetonitrile + 0.1% TFA over 6.80 min. MS (ESI$^+$, $[M]^+$, $C_{64}H_{92}Br_2N_{10}O_7$): 1271.50.

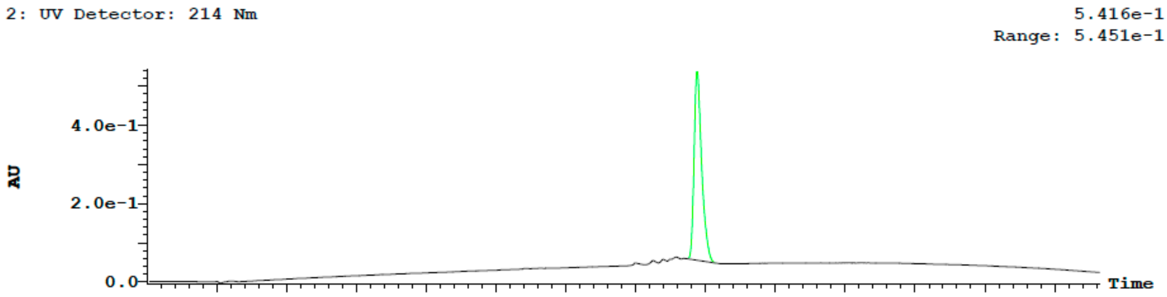

Peak Number	Time	AreaAbs	Area %Total	Height	Mass Found
3	3.94	3.05e+004	100.00	4.86e+005	.00, 1272.00, 1273.00

Peak ID	Time	Mass Found
3	3.95	46,1296.41,425.58,637.41,1274.12

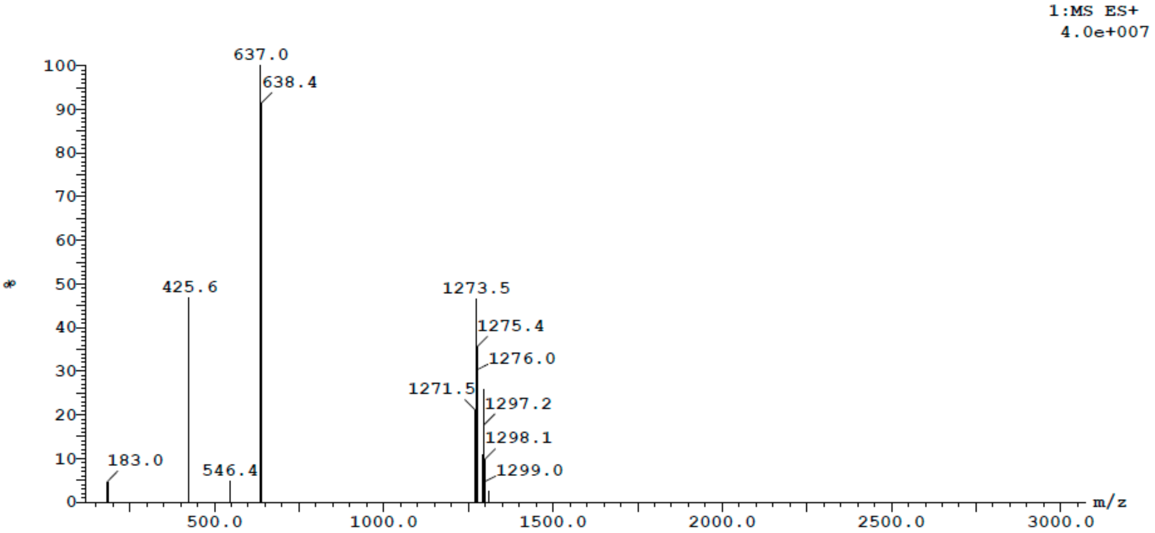

LC / MS trace of MXB10. t_R = 4.22 min. Gradient: 5–95% acetonitrile + 0.1% TFA over 6.80 min. MS (ESI$^+$, [M]$^+$, $C_{67}H_{98}Br_2N_{10}O_7$): 1315.53.

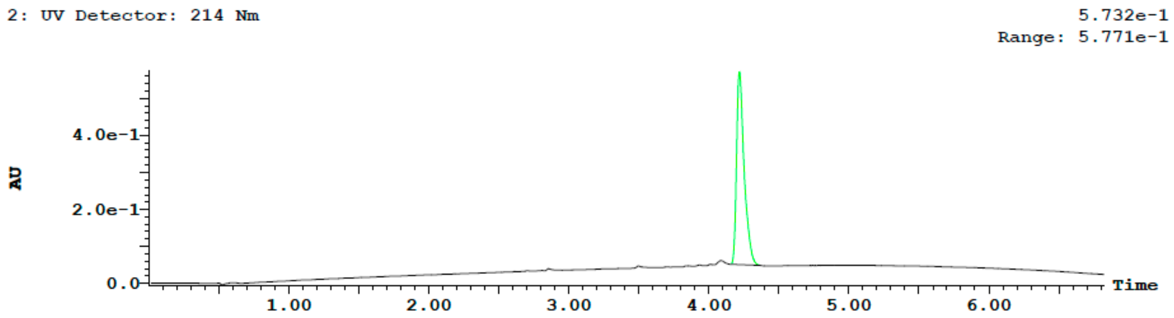

Peak Number	Time	AreaAbs	Area %Total	Height	Mass Found
2	4.22	3.30e+004	100.00	5.22e+005	1314.00, 1315.00

Peak ID	Time	Mass Found
2	4.23	57,1338.34,439.82,658.45,1315.53

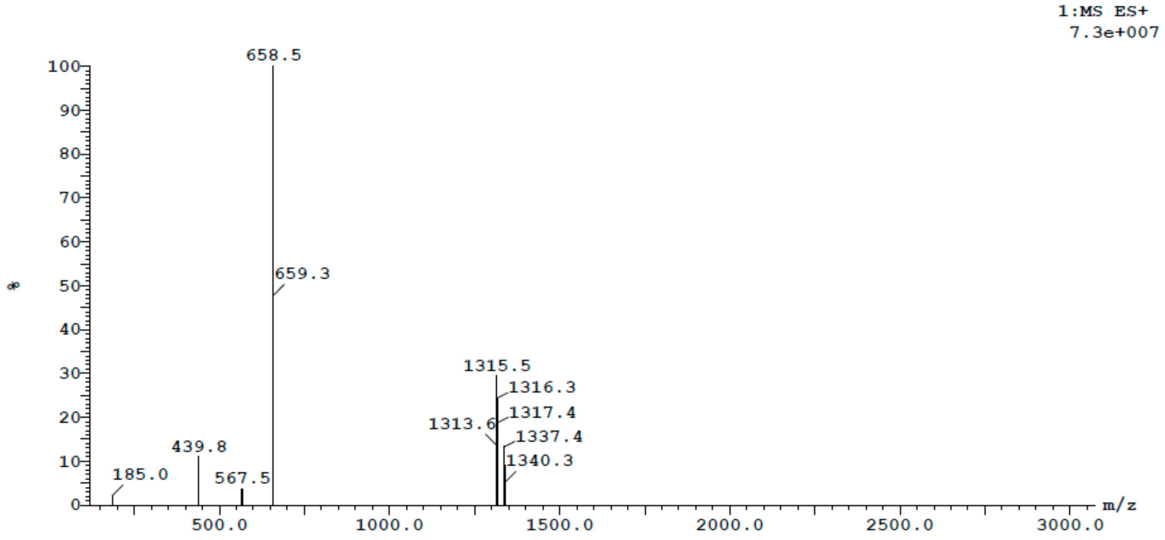

Supplement: Supplementary file 1 [file pharmaceuticals-14-00304-s001.pdf]
